# Supplementary material for: The causal relationship between the human gut microbiota and pyogenic arthritis: a Mendelian randomization study
Source: Front Cell Infect Microbiol. 2024 Nov 26;14:1452480. doi: 10.3389/fcimb.2024.1452480 (PMC11629706; doi:10.3389/fcimb.2024.1452480)
Supplement: Supplementary file 1 [file DataSheet1.zip › mendelian test/ebi-a-GCST90027516.csv_forest.pdf]

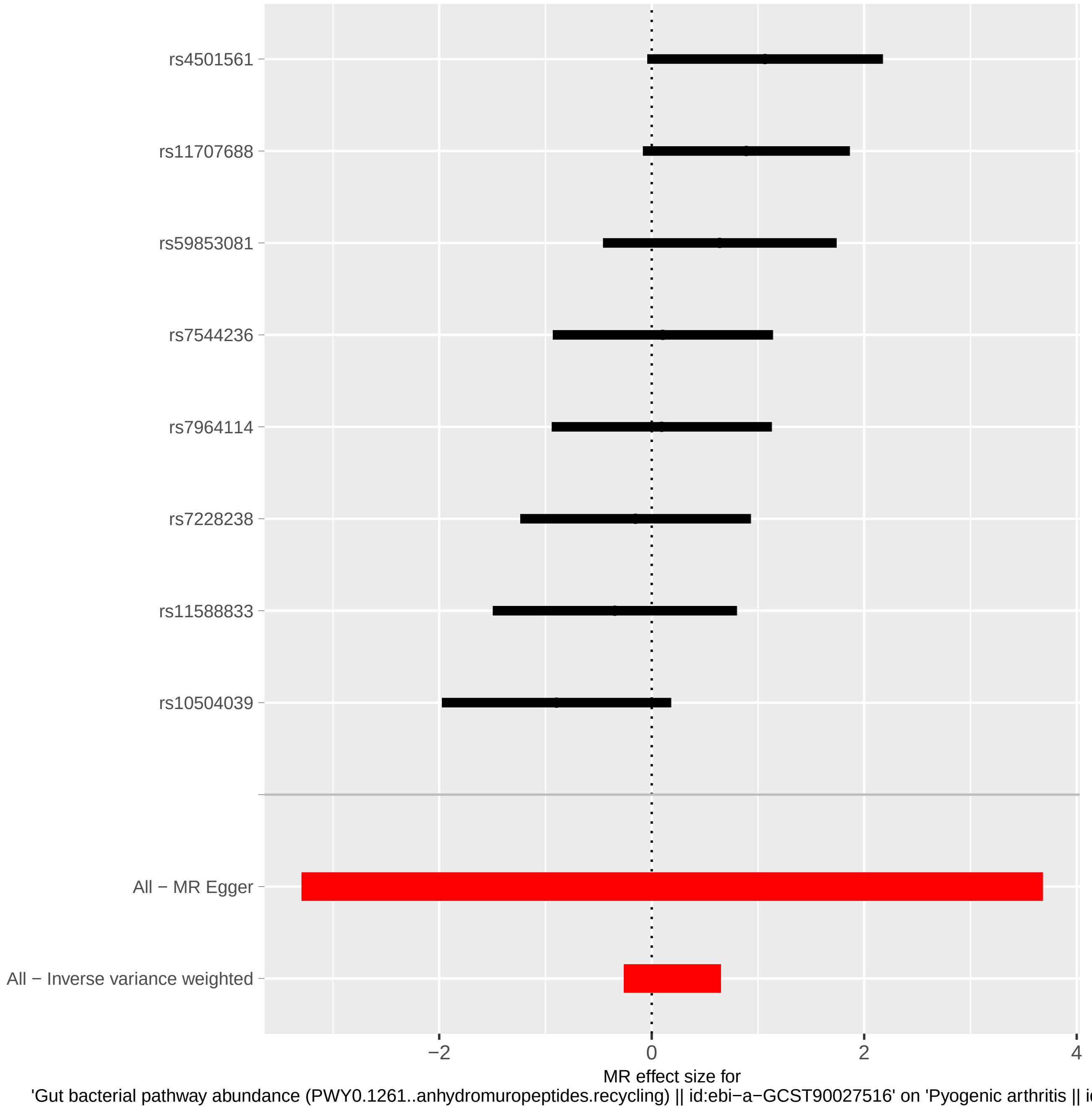
rs4501561
rs11707688
rs59853081
rs7544236
rs7964114
rs7228238
rs11588833
rs10504039
All − MR Egger
All − Inverse variance weighted
−2
0
2
4
MR effect size for
'Gut bacterial pathway abundance (PWY0.1261..anhydromuropeptides.recycling) || id:ebi−a−GCST90027516' on 'Pyogenic arthritis || i
